# Osteocytic vinculin controls bone mass by modulating Mef2c-driven sclerostin expression in mice

**DOI:** 10.1038/s41413-025-00452-x

**Published:** 2025-08-13

**Authors:** Yishu Wang, Jianmei Huang, Sixiong Lin, Lei Qin, Dingyu Hao, Peijun Zhang, Shaochuan Huo, Xuenong Zou, Di Chen, Guozhi Xiao

**Affiliations:** 1 Department of Biochemistry, Homeostatic Medicine Institute School of Medicine Shenzhen Key Laboratory of Cell Microenvironment, Guangdong Provincial Key Laboratory of Cell Microenvironment and Disease Research, Southern University of Science and Technology, Shenzhen, China; 2 The First Affiliated Hospital, Southern University of Science and Technology, Shenzhen, China; 3 Department of Spine Surgery, Orthopedic Research Institute, The First Affiliated Hospital of Sun Yat-sen University, Guangdong Provincial Key Laboratory of Orthopedics and Traumatology, Guangzhou, China; 4 Department of Orthopaedics, The First Affiliated Hospital of Guangzhou Medical University, Guangzhou, China; 5 Department of Orthopedics, Shenzhen Nanshan People’s Hospital, and the 6th Affiliated Hospital of Shenzhen University Medical School, Shenzhen, China; 6 Research Institute, Shenzhen Hospital (Futian) of Guangzhou University of Chinese Medicine, Shenzhen, China; 7 Research Center for Human Tissues and Organs Degeneration, Shenzhen Institutes of Advanced Technology, Chinese Academy of Sciences, Shenzhen, China

**Keywords:** Bone, Calcium and phosphate metabolic disorders

## Abstract

The focal adhesion (FA) is the structural basis of the cell-extracellular matrix crosstalk and plays important roles in control of organ formation and function. Here we show that expression of FA protein vinculin is dramatically reduced in osteocytes in patients with aging-related osteoporosis. Vinculin loss severely impaired osteocyte adhesion and dendrite formation. Deleting vinculin using the mouse 10-kb *Dmp1-Cre* transgenic mice causes dramatic bone loss in the weight-bearing long bones and spine, but not in the skull, in both young and aged mice by impairing osteoblast formation and function without markedly affecting bone resorption. Vinculin loss impairs the anabolic response of skeleton to mechanical loading in mice. Vinculin knockdown increases, while vinculin overexpression decreases, sclerostin expression in osteocytes without impacting expression of Mef2c, a major transcriptional regulator of the *Sost* gene, which encodes sclerostin. Vinculin interacts with Mef2c and retains the latter in the cytoplasm. Thus, vinculin loss enhances Mef2c nuclear translocation and binding to the *Sost* enhancer *ECR5* to promote sclerostin expression in osteocytes and reduces bone formation. Consistent with this notion, deleting *Sost* expression in osteocytes reverses the osteopenic phenotypes caused by vinculin loss in mice. Finally, we find that estrogen is a novel regulator of vinculin expression in osteocytes and that vinculin-deficient mice are resistant to ovariectomy-induced bone loss. Thus, we demonstrate a novel mechanism through which vinculin inhibits the Mef2c-driven sclerostin expression in osteocytes to promote bone formation.

## Introduction

The adult skeleton is a dynamic tissue that undergoes constant bone remodeling, a process during which the osteoclast-mediated bone resorption is closely coupled with the osteoblast-mediated bone formation.^1,2^ Impairment in the bone remodeling process results in metabolic bone diseases, such as osteoporosis characterized with low bone mass and bone fracture.^3,4^ Understanding the mechanisms behind bone remodeling will advance bone biology research and identify new targets for treating metabolic bone diseases.^5^

The focal adhesion (FA), which consists of integrins, extracellular matrix (ECM), cytoskeleton, kindlin-2, talin, vinculin and other proteins, mediates communications between cells and their environment and plays a central role in regulation of the cell-ECM adhesion, migration, differentiation, survival and mechanotransduction.^6–13^ Abnormalities in expression and activation of the FA proteins are involved in the pathogenesis and metastasis of cancers.^14–19^ More recent studies reveal that FA proteins are critical for control of formation and organs and tissues, including skeleton,^19–29^ heart,^30–32^ kidney,^33–35^ pancreatic islets,^36^ adipose tissue,^37^ small intestine,^38^ testicle,^39^ and liver.^40–42^ Vinculin is a key FA protein that is highly concentrated when cells contact one another and the underlying substratum.^43–45^ Vinculin is critical for the FA formation and maintenance. Cells overexpressing vinculin assemble large FAs,^46,47^ whereas vinculin-deficient cells form smaller and fewer FAs.^48,49^ Vinculin exists in two conformations in the cell, i.e., an open, active form and a closed, auto-inhibited state in which the head domain forms extensive interactions with the tail.^50,51^ Mechanical stimulation alters the morphology of vinculin. Vinculin also responds to force and conducts to the cytoskeleton.^52,53^ Previous studies have demonstrated that vinculin is necessary for the development and homeostasis of platelets,^54^ neocortical neurons,^55^ kidney,^35,56^ pancreatic islets,^57,58^ stomach^59,60^ and breast.^61^ However, the function of vinculin in bone has not been explored.

In this study, we demonstrate mice lacking vinculin in the dentin matrix protein 1 (Dmp1)-positive cells (i.e., osteocytes and mature osteoblasts) display a severe bone loss with impaired bone formation, fail to properly respond to mechanical loading, and do not show further bone loss on estrogen deficiency. Vinculin interacts with Mef2c and vinculin loss increases Mef2c nuclear translocation and binding to the *Sost* enhancer *ECR5* to promote sclerostin expression in osteocytes and thereby inhibit bone formation.

## Results

### Vinculin is downregulated in osteocytes in human osteoporotic bones and vinculin loss impairs osteocyte adhesion and dendrite formation

**Fig. 1:**
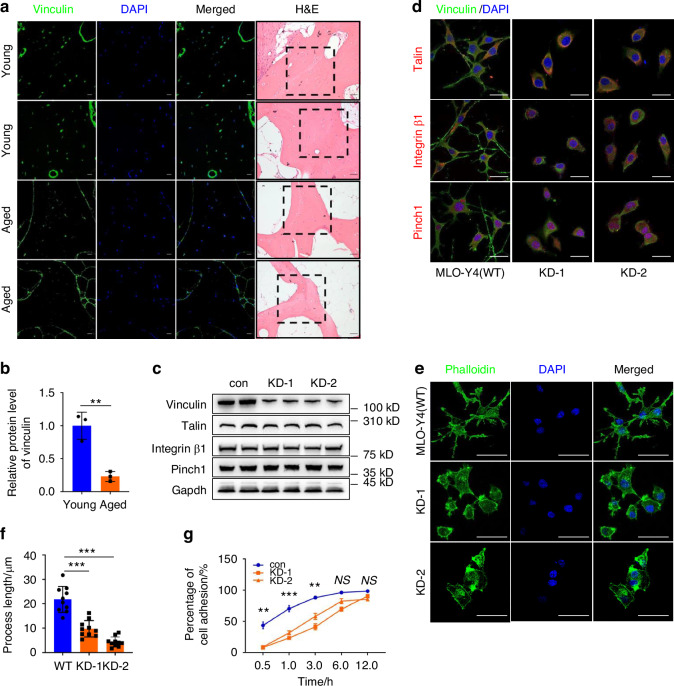
Vinculin expression is drastically reduced in osteocytes in human osteoporotic bones and vinculin loss impairs osteocyte adhesion and dendrite formation. **a**, **b** Immunofluorescence (IF) staining and hematoxylin and eosin (H/E) staining. Sections of female cancellous samples from young (29–32-yrs-old) and aged 78–88-yrs-old) subjects were subjected to H/E and IF staining with a vinculin antibody. Scale bars: 50 μm. Quantitative data (**b**). Results were expressed as mean ± s.d. *n* = 3 biologically independent replicates per group, **P < 0.01 versus young, unpaired two-tailed Student’s *t* test. **c** Western blot analysis. Protein extracts isolated from MLO-Y4 osteocyte-like cells with and without vinculin knockdown (KD) by CRISPR-Cas9 technology were subjected to western blotting using the indicated antibodies. **d** IF staining. MLO-Y4 cells with and without vinculin KD were subjected to IF staining using phalloidin or DAPI, Scale bars, 20 μm. **e**, **f** IF staining. MLO-Y4 cells with and without vinculin KD were subjected to IF staining using the indicated antibodies (**e**). Quantitative data (**f**), Scale bars, 20 μm. **g** Cell adhesion assay. MLO-Y4 cells with and without vinculin KD were seeded in a 96-well plate at a density of 1 × 10^5^ cells/well. The absorbance was measured at the time points of 0.5 h, 1 h, 3 h, 6 h and 12 h, respectively. Results were expressed as mean ± s.d., **P < 0.01, ***P < 0.001 versus controls, unpaired two-tailed Student’s *t* test

As an initial attempt to explore the potential involvement of alteration in expression of FA protein vinculin in the pathogenesis of osteoporosis, human bone tissue samples were collected from young (from 29 to 32 years old) and aged individuals (from 78 to 88 years old) and subjected to immunofluorescence (IF) staining of these bone sections to determine the expression level of vinculin protein. The results showed that osteocytes embedded in the trabecular bone matrix in the young bone samples expressed a high level of vinculin protein, which was drastically decreased in the aged bones (Fig. 1a, b). To gain insights into the function of vinculin in osteocytes, the CRISPR-Cas9 technology was utilized to delete the *Vcl* gene, which encodes vinculin, in MLO-Y4 osteocyte-like cells. During clone selection (from a total of 60 clones), we did not obtain any MLO-Y4 clones with two alleles of the *Vcl* gene deleted. It is possible that they did not grow and died during the selection in culture. We therefore knocked down (KD) vinculin expression by deleting one allele of the *Vcl* gene in MLO-Y4 cells. Note: vinculin KD did not markedly affect the expression levels of other FA proteins, such as integrin β1, kindlin-2, talin and pinch1 (Fig. 1c, d), as measured by IF staining and western blotting analyses. Vinculin KD greatly altered the distribution and orientation and length of filamentous actin (Fig. 1e, f) and impaired plastic plates (Fig. 1g) and Col-I coated (Fig. S1A) cell spreading in MLO-Y4 cells (Fig. 1g).

### Deleting vinculin expression in Dmp1-expressing cells causes a dramatic bone loss in mice

**Fig. 2:**
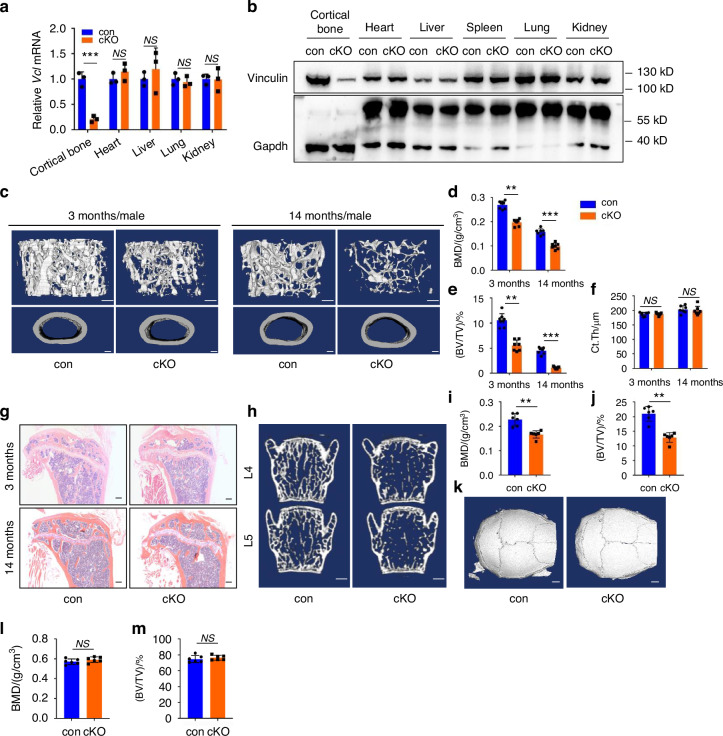
Vinculin loss in osteocytes causes dramatic bone loss in the weight-bearing long bones and spine, but not in the skull. **a** Real-time RT-PCR (qPCR) analyses. Total RNAs isolated from the indicated tissues of 3 mo-old male control (con) and cKO mice were used for qPCR analysis for expression of *Vcl* gene, which was normalized to *Gapdh* mRNA. Results were expressed as mean ± s.d., *n* = 3 biologically independent replicates per group, **P < 0.01 versus controls, unpaired two-tailed Student’s *t* test. **b** Western blot analysis. Protein extracts were isolated from the indicated tissues of the control and cKO mice were subjected to western blotting analysis for vinculin expression. Gapdh was used for loading control. **c** Three-dimensional (3D) reconstruction from micro-computerized tomography (μCT) scans of distal femurs from male control and cKO mice with the indicated ages. Scale bars, 250 μm. **d**–**f** Quantitative analyses of the bone mineral density (BMD), bone volume/tissue volume (BV/TV) and cortical thickness (Ct.Th) of distal femurs from male control and cKO mice with the indicated ages. Results were expressed as mean ± s.d., *n* = 6 biologically independent replicates per group, *P < 0.05, **P < 0.01, ***P < 0.001 versus controls, unpaired two-tailed Student’s *t* test. **g** H/E staining of tibial sections of 3-month-old male control and cKO mice with the indicated ages. **h** 3D reconstruction from μCT scans of spine (L4, L5) from 3-month-old male control and cKO mice. **i**, **j** Quantitative analyses of the bone mineral density (BMD) and bone volume/tissue volume (BV/TV) of spine from male control and cKO mice. Results were expressed as mean ± s.d.*, n* = 6 biologically independent replicates per group, *P < 0.05, **P < 0.01 versus controls, unpaired two-tailed Student’s *t* test. **k** 3D reconstruction from μCT scans of skull. Scale bars, 250 μm. **l**, **m** Quantitative analyses of the BMD and BV/TV of skulls from ma**l**e control and cKO mice. Results were expressed as mean ± s.d., *n* = 6 biologically independent replicates per group, *P < 0.05, **P < 0.01, ***P < 0.001 versus controls, unpaired two-tailed Student’s *t* test

To investigate potential role of vinculin in bone, we deleted its expression in the dentin matrix protein 1 (Dmp1)-expressing cells (primarily osteocytes and mature osteoblasts) by breeding the floxed *Vcl* mice (*Vcl*^*fl/fl*^) with the 10-kb mouse *Dmp1-Cre* transgenic mice (*Dmp1-Cre; Vcl*^*fl/fl*^, referred as cKO hereafter). The results from qRT-PCR and western blotting analyses showed that vinculin mRNA and protein expression was significantly reduced in cKO cortical bones compared to that in control bones (Fig. 2a, b). Micro-computed tomography (μCT) analysis of distal femurs from 3- and 14-month-old male control and cKO mice showed that vinculin loss caused a severe osteopenia at both ages. Vinculin loss significantly decreased the bone mineral density (BMD) (Fig. 2c, d) and bone volume fraction (BV/TV) (Fig. 2c, e) without affecting the cortical thickness (Ct.Th) (Fig. 2f). BMD and BV/TV were also significantly decreased in 6-month-old female cKO mice compared to those in control group (Fig. S1B–E). Despite no marked alteration in Ct.Th between control and cKO mice, three-point bending experiments demonstrated that the mechanical properties of the femur in cKO mice were impaired, with lower ultimate force and whole bone toughness in cKO versus control (Fig. S1F, G). Consistent with results from μCT analyses, results from H/E staining of tibial sections showed much less trabecular bone mass in cKO mice than that in control mice (Fig. 2g). Similar reductions in BMD (Fig. 2h, i) and BV/TV (Fig. 2h, j) were observed in 3-month-old male cKO spine (L4-5) compared to those in control mice (Fig. 2h–j). Note: vinculin loss did not affect the bone mass in skull (Fig. 2k–m).

### Vinculin loss mainly impairs bone formation without markedly impacting bone resorption

**Fig. 3:**
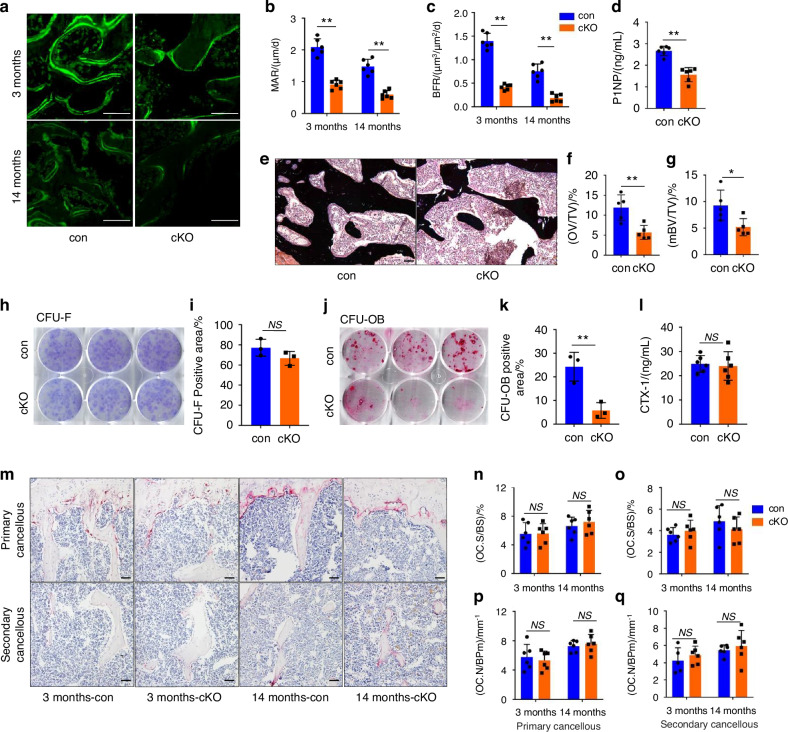
Vinculin loss impairs the osteoblast-mediated bone formation without affecting the osteoclast formation and bone resorption. **a**–**c** Calcein double labeling. Representative images of 3-month-old male control and cKO femur sections (**a**). Sections of non-demineralized femurs of 3-month-old male control and cKO mice were used for measurements of the mineral apposition rate (MAR) and bone formation rate (BFR). Quantitative MAR (**b**) and BFR (**c**) for the metaphyseal trabecular bones of the two genotypes. Scale bar, 50 μm. Results were expressed as mean ± s.d., *n* = 6 biologically independent replicates per group, *P < 0.05, **P < 0.01 versus controls, unpaired two*-*tailed Student’s *t* test. **d** Serum level of procollagen type 1 amino-terminal propeptide (P1NP). Sera harvested from 3-month-old male control and cKO mice were subjected to ELISA assay for P1NP. Results were expressed as mean ± s.d., *n* = 6 biologically independent replicates per gro*up*, **P < 0.01 versus controls, unpaired two-tailed Student’s *t* test. **e**–**g** von Kossa staining. Undecalcified sections of femora from 3-month-old male control and cKO mice were subjected to von Kossa staining (**e**). Quantitative OV/TV (osteoid volume/tissue volume) (**f**) and mBV/TV (mineralized bone volume/tissue volume) (**g**) data for the cancellous bones from distal femora were measured by bone morphometry. Scale bar, 50 μm. Results were expressed as mean ± s.d., *n* = 5 biologically independent replicates per group, *P < 0.05, **P < 0.01 versus controls, unpaired two-tailed Student’s *t* test. **h**, **i** Colony forming unit-fibroblast (CFU-F) assays. Bone marrow nucleated cells from 3-month-old male control and cKO mice were seeded in a 6-well plate with a cell density of 2 × 10^6^ per well and cultured using the Mouse MesenCult proliferation kit (CFU-F assay) for 14 days, followed by Giemsa staining (**h**). Quantitative data (**i**). **j**, **k** Colony forming unit-osteoblast (CFU-OB) assays. Bone marrow nucleated cells from 3-month-old male control and cKO mice were seeded in a 6-well plate with a cell density of 4 × 10^6^ per well and cultured in osteoblast differentiation medium (complete α-MEM containing 50 μg/mL L-ascorbic acid and 2.0 mmol/L β-glycerophosphate) for 21 d and media were changed every 48 h, followed by alizarin red staining (**j**). Quantitative data (**k**). **l** Serum level of collagen type I cross-linked C-telopeptide (CTX). Sera collected from 3-months-old male control and cKO mice were subjected to ELISA assay for CTX. Results were expressed as mean ± s.d., *n* = 6 biologically independent replicates per group, *P < 0.05 versus controls, unpaired two-tailed *t* test. **m**–**q** Tartrate-resistant acid phosphatase (TRAP) staining. Tibial sections of 3-month-male control and cKO mice were used for TRAP staining (**m**). Osteoclast surface/bone surface (Oc.S/BS) (**n**, **o**) and osteoclast number/bone perimeter (Oc.N/BPm) (**p**, **q**) of primary and secondary cancellous bones were measured using Image-Pro Plus 7.0. Scale bar, 50 μm. Results were expressed as mean ± s.d., *n* = 7 biologically independent replicates per group, *P < 0.05 versus controls, unpaired two-tailed Student’s *t* test

To investigate mechanism through which vinculin loss causes a low bone mass, the formation and function of osteoblasts and osteoclasts in control and cKO long bones were determined. Results from the double calcein labeling experiments showed that the mineralization apposition rate (MAR) and bone formation rate (BFR) in the femur metaphyseal cancellous bones from 3-month-old male cKO mice were significantly decreased compared to those in control mice (Fig. 3a–c). Consistent findings in cortical bone indicated that 3-month-old cKO mice had decreased MAR and BFR compared to the control group (Fig. S2A–C). The serum level of the procollagen type 1 amino-terminal propeptide (P1NP), a marker of in vivo bone formation,^62^ was significantly lower in cKO mice than that in control mice (Fig. 3d). To determine the effects of vinculin loss on the osteoid production and mineralization, we performed the von Kossa staining of femur sections of control and dKO mice and found that the osteoid volume/tissue volume (OV/TV) and mineralized bone volume/tissue volume (mBV/TV) were decreased in cKO mice relative to those in control mice (Fig. 3e–g). We next performed the colony forming unit-fibroblast (CFU-F) assay and colony forming unit-osteoblast (CFU-OB) assay. The results showed that the number of CFU-OB, but not that of CFU-F, was reduced in cKO group compared to that in control group (Fig. 3h–k). Osteoclast formation and bone-resorbing activity were further measured in control and cKO mice. The serum levels of collagen type I cross-linked C-telopeptide (CTX), degradation products from type I collagen during in vivo osteoclastic bone resorption,^62^ were not significantly different between the two genotypes (Fig. 3l). Results from TRAP staining of tibial sections showed that the osteoclast surface/bone surface (Oc.S/BS) and osteoclast number/bone perimeter (Oc.Nb/BPm) in both primary and secondary cancellous bones were not markedly altered in cKO mice relative to those in control mice (Fig. 3m–q). Vinculin loss led to changes in the number and length of osteocyte dendrites. Loss of vinculin caused alterations in the number and length of osteocyte dendrites (Fig. S3A–C).

### Vinculin loss increases, while vinculin overexpression decreases, sclerostin expression without affecting the level of Mef2c protein in osteocytes

**Fig. 4:**
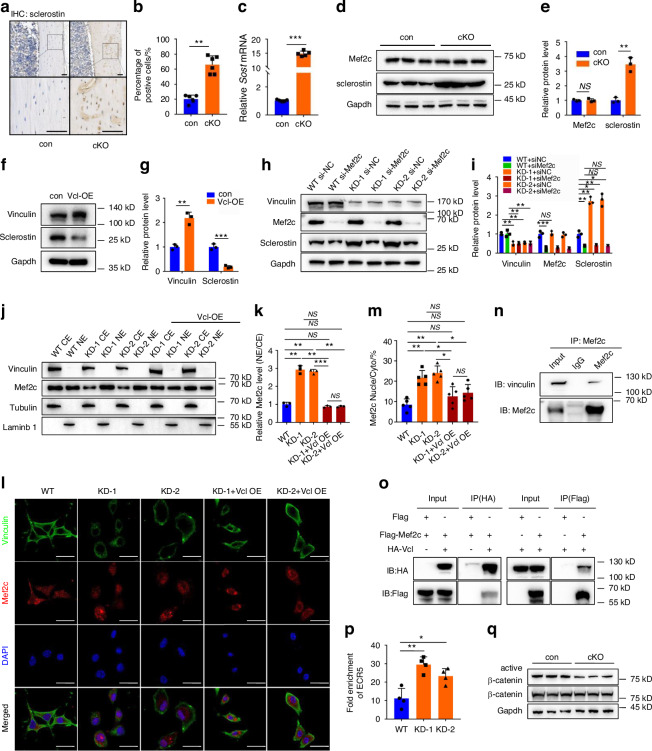
Vinculin interacts with Mef2c and vinculin knockdown increases Mef2c nuclear translocation and binding to the *Sost* enhancer *ECR5* to promote sclerostin expression in osteocytes. **a**, **b** Immunohistochemical (IHC) staining. Tibial sections of 3-month-old male control and cKO mice were stained with an anti-sclerostin antibody. Quantitative data (**b**). *n* = 5 biologically independent replicates per group, **P < 0.01, versus controls, unpaired two-tailed Student’s *t* test. **c** qPCR analyses. Total RNA isolated from cortical bone of 3-month-old male mice was used for qPCR analysis for expression of *Sost* gene, which was normalized to *Gapdh* mRNA. Results were expressed as mean ± s.d., *n* = 5 biologically independent replicates per group, ***P < 0.001 versus controls, unpaired two*-*tailed Student’s *t* test. **d**, **e** Western blot analyses. Protein extracts isolated from cortical bones of 3 mo-old male mice were subjected to western blotting using the indicated antibodies. Results were expressed as mean ± s.d., *n* = 3 biologically independent replicates per group, **P < 0.01 versus controls, unpaired two-tailed Student’s *t* test. **f**, **g** Vinculin overexpression. Protein extracts isolated from MLO-Y4 cells transfected with control or vinculin-expressing plasmid were subjected to western blotting using the indicated antibodies. Results were expressed as mean ± s.d., **P < 0.01, ***P < 0.001 versus veh, unpaired two-tailed Student’s *t* test. **h**, **i** Whole cell extracts from MLO-Y4 cells with and without vinculin or Mef2c siRNA knockdown (KD) were subjected to western blotting using the indicated antibodies. Results were expressed as mean ± s.d., *P < 0.05, **P < 0.01 versus veh, two-way ANOVA. **j**, **k** Mef2c nuclear translocation. Cytoplasmic (CE) and nuclear extracts (NE) from MLO-Y4 cells with and without vinculin KD and with and without vinculin overexpression (OE) were subjected to western blotting with the indicated antibody. Quantitative data (**k**). Results were expressed as mean ± s.d., *n* = 3 biologically independent replicates per group, **P < 0.01, ***P < 0.001 versus controls, unpaired two-tailed Student’s *t* test. **l** Vinculin-Mef2c colocalization. MLO-Y4 cells treated as in (**h**) were subjected to IF staining with the indicated antibody. Scale bars: 50 μm. Quantitative data (**m**). **n**, **o** Co-immunoprecipitation (co-IP) assay. Protein extracts from MLO-Y4 cells with and without overexpression of HA-vinculin and Flag-Mef2c were incubated with the indicated antibodies or IgG, and the immunocomplexes were separated by SDS-PAGE, followed by western blotting with the indicated antibodies. **p** CHIP-qPCR analysis. The expression of *Sost* enhancer *ECR5* combined with Mef2c antibody, which was normalized to IgG. Results were expressed as mean ± s.d., *P < 0.05, **P < 0.01 versus control, unpaired two-tailed Student’s *t* test. **q** Western blot analyses. Protein extracts isolated from BMSC cultures of 3-month-old male control and cKO mice were subjected to western blotting using the indicated antibodies

Sclerostin is encoded by *Sost* and mainly secreted by osteocytes. Sclerostin decreases osteoblast and bone formation via inhibiting the Wnt/β-catenin signaling. Sclerostin loss or pharmacological neutralization greatly increases bone formation and bone mass.^63^ Because the osteoblast and bone formation was severely impaired by vinculin loss in the cKO mice, as demonstrated above, we next determined whether vinculin loss increased sclerostin production in mice. The cKO mice showed significantly increased levels of sclerostin mRNA and protein in cortical osteocytes, as evidenced by IHC, RT-qPCR, and western blotting analyses. (Fig. 4a–e). The findings in trabecular bones were consistent with those in cortical bones, with more sclerostin-positive cells observed in cKO mice relative to the control group (Fig. S4A, B). Note: the loss of vinculin did not significantly impact the Mef2c protein level in bone extracts (Fig. 4d, e), a major transcriptional regulator of the *Sost* gene. In contrast, overexpression of vinculin decreased the level of sclerostin in MLO-Y4 cells (Fig. 4f, g).

### Vinculin interacts with Mef2c and vinculin loss enhances Mef2c nuclear translocation and binding to the *Sost* enhancer *ECR5* in osteocytes

The results showed that siRNA knockdown (KD) of Mef2c in MLO-Y4 cells reversed the sclerostin upregulation caused by vinculin loss (Fig. 4h, i). Furthermore, reduced levels of cytoplasmic Mef2c and increased levels of nuclear Mef2c were observed in MLO-Y4 cells with vinculin knockdown, and overexpression of vinculin displayed opposite results (Fig. 4j, k). IF staining revealed that vinculin and Mef2c colocalized in MLO-Y4 cells and that vinculin KD increased Mef2c nuclear translocation in these cells, which was reversed by vinculin overexpression (Fig. 4l, m). Results from co-immunoprecipitation (Co-IP) experiments demonstrated interactions of endogenous or overexpressed vinculin and Mef2c proteins in MLO-Y4 cells (Fig. 4n, o). Results from ChIP assay using the MLO-Y4 cells revealed that vinculin KD dramatically increased the binding of Mef2c to the *Sost* enhancer *ECR5* (Fig. 4p). Consistent with the fact that sclerostin is a potent inhibitor of the Wnt/β-catenin pathway, the level of active β-catenin, but not that of its total protein, was dramatically reduced in cKO BMSC cultures relative to that in control BMSC cultures (Fig. 4q).

### Deleting *Sost* expression in Dmp1-expressing cells reverses the osteopenic phenotypes induced by vinculin loss in mice

**Fig. 5:**
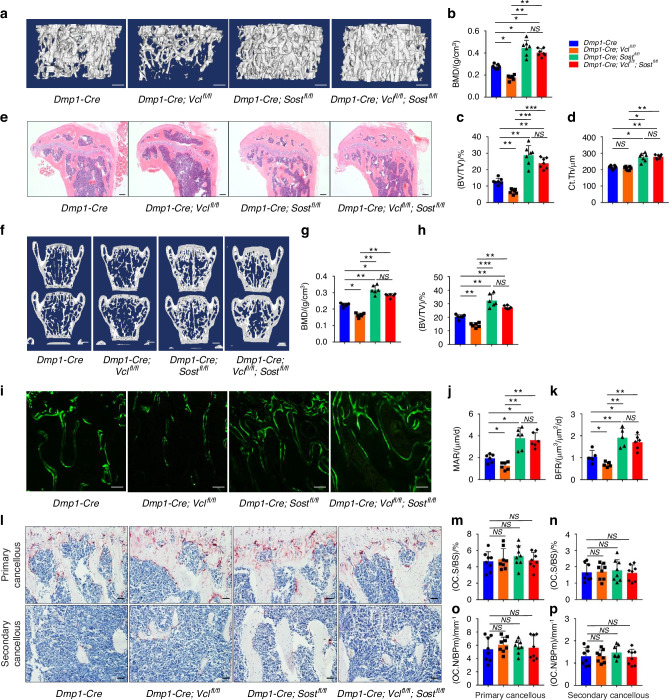
Deleting sclerostin in Dmp1-expressing cells reverses the osteopenia induced by vinculin loss in mice. **a** 3D reconstruction from µCT scans of tibia from 3-month-old male mice with indicated genotype. Scale bar, 250 μm. **b**–**d** Quantitative analyses of BV/TV Ct.Th and BMD. Results were expressed as mean ± s.d., *n* = 7 biologically independent replicates per group, *P < 0.05, **P < 0.01, ***P < 0.001 versus controls, unpaired two-tailed Student’s *t* test. **e** H/E staining of tibial sections of 3-month-old with indicated genotype. Scale bar, 200 μm. **f** 3D reconstruction from μCT scans of spine (L4, L5) from 3-mo-old male mice with indicated genotype. **g**, **h** Quantitative analyses of bone mineral density (BMD) and bone volume/tissue volume (BV/TV) of spine. Results were expressed as mean ± s.d., *n* = 6 biologically independent replicates per group, *P < 0.05, **P < 0.01, ***P < 0.001 versus controls, unpaired two-tailed Student’s *t* test. **i**–**k** Calcein double labeling. Representative images of femur sections (**i**). Sections were used for measurements of mineral apposition rate (MAR). Quantitative MAR data for metaphyseal trabecular bones (**j**) and bone formation rate (BFR) (**k**). Scale bar, 50 μm. **l**–**p** Tartrate-resistant acid phosphatase (TRAP) staining. Tibial sections of 3-month-male mice were used for TRAP staining with indicated genotype (**l**), osteoclast surface/bone surface (Oc.S/BS) (**m**, **n**) and osteoclast number/bone perimeter (Oc.N/BPm) (**o**, **p**) of pri**m**ary and secondary cancellous bones were measured using Image-Pro Plus 7.0. Scale bar, 50 μm. Results were expressed as mean ± s.d., *n* = 6 biologically independent replicates per group, *P < 0.05 versus controls, unpaired two-tailed Student’s *t* test

Above results suggest that the sclerostin upregulation by vinculin loss may play a role in causing the bone loss in cKO mice. To determine if this was the case, the effects of Sost deletion in Dmp1-expressing cells on the osteopenic phenotypes caused by vinculin loss were next determined in mice. We bred the *Dmp1-Cre; Vcl*^*fl/fl*^ (cKO) mice with *Sost*^*fl/fl*^ mice and generated *Dmp1-Cre; Vcl*^*fl/fl*^*; Sost*^*fl/fl*^ and *Dmp1-Cre; Sost*^*fl/fl*^ mice. μCT analysis was performed on the femurs with indicated groups (Fig. 5a). As expected, *Sost* loss increased the BMD, BV/TV and Ct.Th (Fig. 5b–d). Importantly, *Sost* deletion largely reversed the osteopenia of cKO mice (Fig. 5b, c). H/E staining of the tibial sections revealed that *Sost* ablation reversed the low bone mass in vinculin-deficient mice (Fig. 5e). *Sost* inactivation similarly reversed the osteopenic phenotypes in spine (L4-L5) in vinculin cKO mice (Fig. 5f–h). Furthermore, the mechanical properties of femurs, including ultimate force and whole bone toughness, were largely recovered by *Sost* ablation in vinculin-deficient mice (Fig. S5A, B).

Because it is known that sclerostin reduces bone formation by inhibiting Wnt/β-catenin pathway, the bone-forming activity of osteoblasts in vivo was measured through double calcein labeling experiments. As expected, we observed significant increases in the MAR and BFR after *Sost* inactivation (Fig. 5i–k). Importantly, deleting *Sost* expression largely restored the impairment in bone formation in cKO mice (Fig. 5i–k). Results from the TRAP staining of tibial sections indicated no marked differences in osteoclast formation between the control and cKO mice with and without *Sost* deletion (Fig. 5l–p).

### Vinculin loss greatly impairs the anabolic responses of skeleton to mechanical loading in mice

**Fig. 6:**
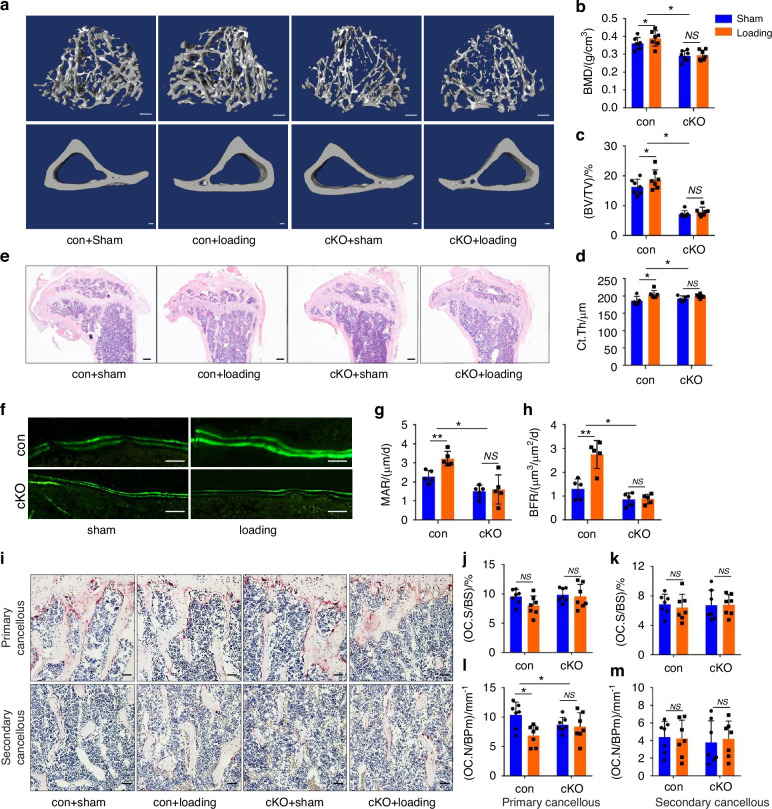
Vinculin deletion reduces mechanical loading-stimulated bone formation in mice. **a** 3D reconstruction from μCT scans of tibiae from 3-month-old male control and cKO mice treated with or without tibia loading experiment. Scale bar, 100 μm. **b**–**d** Quantitative analyses of BMD, BV/TV and Ct.Th. Results were expressed as mean ± s.d., *n* = 7 biologically independent replicates per group, *P < 0.05, **P < 0.01 versus controls, two-way ANOVA. **e** H/E staining of tibial sections of 4-month-old control and cKO mice were treated with or without tibia loading experiment. Scale bar, 200 μm. **f**–**h** Calcein double labeling. Representative images of tibial sections (**f**). Sections were used for measurements of MAR and BFR for metaphyseal trabecular bones. Scale bar, 50 μm. Results were expressed as mean ± s.d., *n* = 5 biologically independent replicates per group, *P < 0.05, **P < 0.01 versus controls, two-way ANOVA. **i**–**m** TRAP staining. Tibial sections of 4-month**-**male control and cKO mice were used for TRAP staining (**i**). Oc.S/BS) (**j**, **k**) and Oc.N/BPm (**l**, **m**) of pr**i**mary and secondary cancellous bones were measured using Image-Pro Plus 7.0. Scale bar, 50 μm. Results were expressed as mean ± s.d., *n* = 6 biologically independent replicates per group, *P < 0.05 versus controls, two-way ANOVA

It is now widely believed that osteocytes embedded in the mineralizing matrix are the major mechanosensors of bone.^10,64^ The involvement of vinculin in bone mechanotransduction was next examined through tibial loading experiments in control and cKO mice.^65^ Results from μCT analyses showed that 2 weeks’ mechanical loading caused significant increases in the BMD, BV/TV and Ct.Th in control tibiae, which were essentially abolished in cKO tibiae (Fig. 6a–d). Results from H/E staining of the tibial sections showed that mechanical loading markedly increased the trabecular volume in control but not cKO mice (Fig. 6e). Mechanical loading significantly increased the values of MAR and BFR in control but not cKO tibiae (Fig. 6f–h). Finally, tibial loading decreased the Oc.N/BPm, but not the Oc.S/BS, in control mice, which was lost in cKO mice (Fig. 6i–m).

### Estrogen regulates vinculin expression in osteocytes and vinculin-deficient mice are resistant to ovariectomy-induced bone loss in mice

**Fig. 7:**
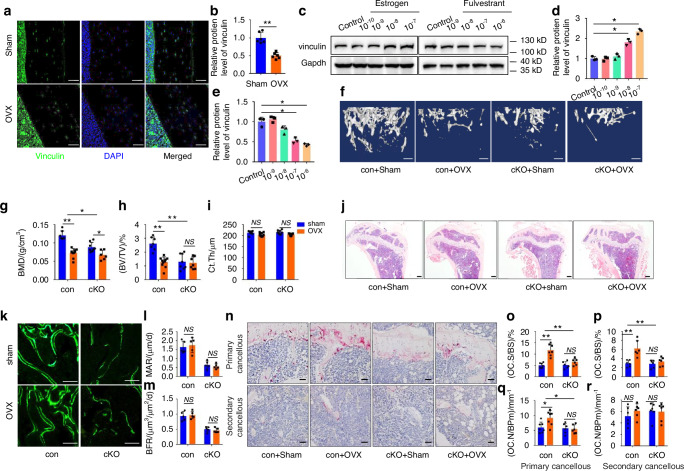
Estrogen regulates vinculin expression in osteocytes and vinculin cKO mice are resistant to OVX-induced bone loss. **a**, **b** IF staining. Tibial sections of sham and OVX mice with an anti-vinculin antibody, Scale bars: 50 μm. Qauntitative data (**b**). *n* = 6 biologically independent replicates per group, **P < 0.01, versus controls, two-way ANOVA. **c**–**e** Western blot analysis. MLO-Y4 cells were treated with increasing concentration of estrogen or fulvestrant (an estrogen receptor antagonist) for 24 h. Gapdh was used as a loading control. Quantitative data from three biologically independent replicates. Results were expressed as mean ± s.d., *P < 0.05 versus control. **f** 3D reconstruction from μCT scans of femurs from 5-month-old control and cKO female mice performed with sham or OVX surgeries. Scale bar, 250 μm. **g**–**i** Quantitative analyses of the BMD BV/TV and Ct.Th. Results were expressed as mean ± s.d., *n* = 6 biologically independent replicates per group, *P < 0.05, **P < 0.01 versus controls, two-way ANOVA. **j** H/E staining of tibial sections. Scale bar, 200 μm. **k**–**m** Calcein double labeling. Representative images of 3-month-old male femur sections (**k**). Sections of non-demineralized femurs were used for measurements of MAR and BFR for the metaphyseal trabecular bones. Scale bar, 50 μm. Results were expressed as mean ± s.d., *n* = 6 biologically independent replicates per group, *P < 0.05 versus controls, two-way ANOVA. **n**–**r** TRAP staining. Tibial sections of 3-month-male control and cKO mice were used for TRAP staining (**n**). Oc.S/BS (**o**, **p**) and Oc.N/BPm (**q**, **r**) of primary and secondary cancellous bones were measu**r**ed using Image-Pro Plus 7.0. Scale bar, 50 μm. Results were expressed as mean ± s.d., *n* = 6 biologically independent replicates per group, *P < 0.05, **P < 0.01 versus controls, two-way ANOVA

Vinculin expression in osteocytes was further determined in mice subjected to ovariectomy (OVX), a procedure that mimics postmenopausal osteoporosis in humans. Surprisingly, we found that the expression level of vinculin protein was strikingly decreased in osteocytes embedded in the bone matrix in OVX mice (Fig. 7a, b). Estrogen dose-dependently increased the level of vinculin in MLO-Y4 cells. (Fig. 7c, d). In contrast, fulvestrant, an estrogen receptor antagonist, decreased the vinculin protein level in MLO-Y4 cells (Fig. 7c, e). The effects of OVX on bone mass reduction were compared between control and vinculin cKO mice. To this end, 3-month-old control and vinculin cKO female mice were subjected to sham or OVX surgery, as we previously described.^23^ As expected, a decrease in bone mass was observed in the control mice with OVX two months after surgery. Shockingly, the OVX-induced trabecular bone loss was dramatically reduced (BV/TV) or completely abolished (BMD) in cKO mice (Fig. 7f–i). While OVX did not markedly impact the MAR and BFR in both control and cKO mice (Fig. 7k–m), it caused dramatic increases in the Oc.S/BS and Oc.N/BPm in control mice, which were lost in vinculin cKO mice (Fig. 7n–r). OVX increased the number of Rankl-expressing osteocytes and decreased that of Opg-expressing osteocytes in control mice; the magnitudes of these changes were reduced in cKO mice (Fig. S6A–D).

## Discussion

The main findings of the present study are summarized as follows: First, the expression of FA protein vinculin is drastically reduced in osteocytes in patients with age-related osteoporosis and in mice with estrogen deficiency. Second, deleting vinculin in Dmp1-expressing cells, i.e., primarily osteocytes and mature osteoblasts, causes a severe bone loss in both young and aged mice. Third, vinculin deletion impairs the bone mechanotransduction in mice. Fourth, vinculin expression in osteocytes is critically involved in estrogen control of bone mass in mice. Finally, vinculin is required for formation of the osteocyte dendrites (Fig. S3A–C) and inhibits sclerostin expression to promote bone formation.^66^

In this study, we demonstrate that osteocytic vinculin is critical for maintaining bone homeostasis. Thus, vinculin loss in osteocytes and mature osteoblasts dramatically decreased the values of serum P1NP, BFR, MAR, OV/TV and mBV/TV, all osteoblastic parameters. These impairments lead to reduced bone formation and severe osteopenia. Of note, vinculin loss does not markedly alter osteoclast formation (Oc.S/BS and Oc.N/BPm) and bone resorption (serum CTX-1). These results on osteoclast formation are similar to those in mice lacking the other FA proteins pinch1/2 in Dmp1-expressing cells,^29^ but in contrast to those in mice with deletion of FA protein kindlin-2 in the same cell type, which displayed increased osteoclast formation and bone resorption in part due to enhanced Rankl production in osteocytes. While it was reported that the 10-kb *Dmp1-Cre* targeted bone and probably other tissues, such as muscle,^67^ our previous study demonstrated that there was no difference observed in the level of kindlin-2 protein in the muscles of mice lacking kindlin-2 in Dmp1-expressing cells (*Dmp1-Cre; Kindlin-2*^*fl/fl*^) compared to control group.^22^

Our findings from this study strongly suggest that vinculin inhibits sclerostin expression in osteocytes and mature osteoblasts to promote the osteoblast-mediated bone formation. Our in vitro and in vivo results demonstrate that vinculin-deficient osteocytes produce a large amount of sclerostin protein, which inhibits binding of Wnt ligands to the LRP5/6 receptors and, thereby, the Wnt/β-catenin signaling. The latter is a major player in control of the bone mass accrual in vertebrates. Consistently, vinculin loss reduces the level of active β-catenin protein and CFU-OB formation in bone marrows and bone formation in mice. Of particular significance, deleting *Sost* expression in Dmp1-expressing cells essentially reverses the low bone mass caused by vinculin loss. Thus, we provide important genetic evidence that FA protein vinculin plays a critical role in promoting bone formation by facilitating the Wnt/β-catenin signaling in osteoblastic cells.

At the molecular level, we provide intriguing evidence that vinculin loss promotes *Sost* expression through, at least in part, Mef2c, a major transcriptional regulator of the *Sost* gene.^68,69^ Importantly, upregulation of sclerostin expression induced by vinculin loss is largely reversed by Mef2c KD in osteocytes. While vinculin loss does not alter the expression level of total Mef2c protein in osteocytes, it increases the Mef2c nuclear translocation and binding to the *Sost* enhancer *ECR5*. It is known that Mef2c binding to this enhancer is critical for *Sost* expression in osteocytes.^70^ Our results suggest that binding of vinculin to Mef2c prevents the Mef2c nuclear translocation by retaining it in the cytoplasm.

While estrogen deficiency causes the postmenopausal osteoporosis, its mechanism(s) remain incompletely understood. Results from the current study reveal that OVX mice express an extremely low level of vinculin protein in osteocytes in bone. Mice lacking vinculin in osteocytes are resistant to OVX-induced bone loss. Estrogen increases vinculin expression in osteocytes. Collectively, these results suggest that estrogen signaling favors bone formation by in part inducing vinculin expression in osteocytes. This knowledge improves our understanding of the molecular mechanism(s) underlying the postmenopausal osteoporosis. An alternative possibility is that the low trabecular bone mass in female mice with osteocyte-specific vinculin deficiency may already reach a minimal threshold, thereby preventing further bone loss.

Mechanical force is the most potent anabolic stimulus for bone formation. Osteocytes, the most abundant cells embedded in the mineralizing matrix in bone, are now believed to be the major sensor and mediator of mechanotransduction in bone.^10^ Results from this study demonstrate that vinculin expression in osteocytes is essential for skeletal response to mechanical loading to increase bone formation. It should be noted that loss of other FA proteins kindlin-2 or pinch in osteocytes caused a similar defect in the anabolic effects of mechanical loading on bone in mice.^24,29^ Collectively, these results suggest a critical role of the FA signaling pathway in mediation of the mechanotransduction in bone. Notably, previous studies demonstrate that mechanical force promotes vinculin activation through conformational changes and a loss of tension causes vinculin to be rapidly inactivated.^71^

It should be noted that vinculin loss causes a dramatic bone loss in the weight-bearing long bones, but not in non-weight bearing skull. The underlaying molecular mechanism(s) are unknown but deserves further investigation.

**Fig. 8:**
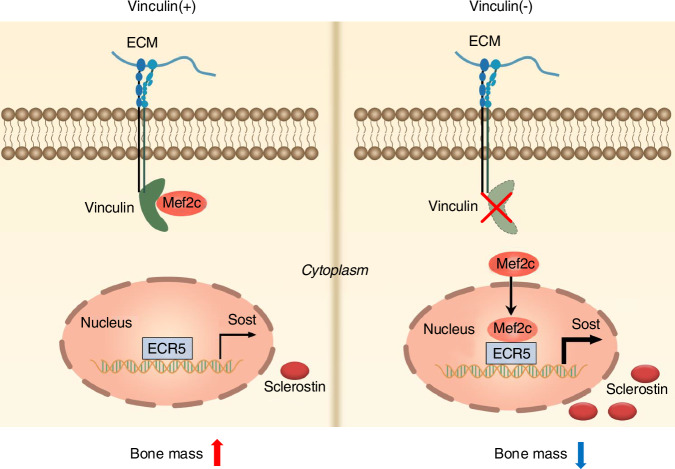
Working model. In osteocytes, vinculin normally binds to transcription factor Mef2c in the cytoplasm and thereby blocks the nuclear translocation of Mef2c and inhibits *Sost* gene expression, thus activating the Wnt/β-catenin pathway and promoting bone formation and bone mass. In the absence of vinculin, Mef2c is translocated into the nuclei in osteocytes where it activates *Sost* expression, thus inhibiting the Wnt/β-catenin pathway and reducing bone formation and bone mass

Finally, we propose a working model regarding how FA protein vinculin expression in osteocytes embedded in the bone matrix controls bone formation (Fig. 8). In young and adult animals, vinculin is highly expressed in osteocytes where it, probably together other FA proteins, such as talin and actin, forms a complex with Mef2c in the cytoplasm. Complex formation prevents the nuclear translocation of Mef2c, leading to reduced expression and production of sclerostin and, thereby, increased bone formation. In contrast, in the presence of vinculin deficiency in osteocytes, which is related to aging and estrogen deficiency, the nuclear translocation of Mef2c and its binding to the *Sost* enhancer *ECR5* are enhanced, thus stimulating Sost expression and inhibiting bone formation. In summary, in this study, we demonstrate that vinculin plays an important role in control of bone formation and may be a useful target for the prevention and treatment of aging and estrogen deficiency induced osteoporosis.

## Materials and methods

### Animal studies

*Dmp1-Cre* mice were generously provided by Dr. Jian Q Feng (Baylor College of Dentistry).^72^ *Vcl*^*fl/fl*^ mice were purchased from the Jackson Laboratory. The experimental animals used current studies were obtained using a 2-step breeding strategy. Homozygous *Vcl*^*fl/fl*^ mice were first crossed with *Dmp1-Cre* transgenic mice to generate *Dmp1-Cre*; *Vcl*^*fl/+*^ mice. These offsprings were then crossed with *Vcl*^*fl/fl*^ to generate *Dmp1-Cre*; *Vcl*^*fl/fl*^ mice. The generation of the *Dmp1-Cre*; *Vcl*^*fl/fl*^; *Sost*^*fl/fl*^ mice was as follow: *Dmp1-Cre*; *Vcl*^*fl/fl*^ mice were first crossed with *Sost*^*fl/fl*^ mice to generate *Dmp1-Cre*; *Vcl*^*fl/+*^; *Sost*^*fl/+*^ mice. These offspring were then intercrossed with *Vcl*^*fl/fl*^; *Sost*^*fl/fl*^ mice to generate the *Dmp1-Cre*; *Vcl*^*fl/fl*^; *Sost*^*fl/fl*^ mice and other genotypes. In vivo tibial loading experiments were performed as previously described.^24^ All animal experiments in this study were approved by the Institutional Animal Care and Use Committees (IACUC) of the Southern University of Science and Technology.

### Tissue samples

Human cancellous tissues were collected from the Shenzhen Hospital of Guangzhou University of Chinese Medicine. Written informed consent was obtained from all patients. The study has been approved by the Shenzhen Hospital of Guangzhou University of Chinese Medicine, China (NO. GZYLL(KY)-2022-027). Specimens were collected and stored in formaldehyde after surgery.

### Micro-computerized tomography (μCT) analysis

Fixed non-demineralized femur, tibia, spine or skull were used for μCT analysis in the Experimental Animal Center of Southern University of Science and Technology using a Bruker μCT (SkyScan 1276 Micro-CT, Bruker MicroCT, Kontich, Belgium) following the standards of techniques and terminology recommended by the American Society for Bone and Mineral Research (ASBMR).^73^

### Histological evaluation and bone histomorphometry

For histology, bone tissues were fixed in 4% paraformaldehyde (PFA) at 4 °C for 24 h, decalcified in 10% EDTA (pH 7.4) for 21 d, and embedded in paraffin after dehydration. Bone sections were used for hematoxylin and eosin (H/E) and tartrate-resistant acid phosphatase (TRAP) staining using our standard protocols.^29,74^

### Immunohistochemistry (IHC) and immunofluorescence (IF) staining

For IHC staining, 5-μm sections were stained with antibodies or control IgG using the EnVision+System-HRP (DAB) kit (Dako North America Inc, Carpinteria, CA, USA) as previously described.^22^ IF staining of bone sections and MLO-Y4 cells was performed as we previously described.^36^

### Femur three-point bending

The three-point bending test was conducted with ElectroForce (Bose ElectroFore 3200; EndureTEC Minnetonka, MN, USA) as we previously described.^24^ After removing the soft tissues, femurs were rapidly transferred into 1xPBS and stored at 4 °C until the time of the bending test. A strength assessment was carried out at the mid-section of the femurs, utilizing a single stop setting with a continuous displacement rate of 0.05 mm/s. The strength test method employed a ramp waveform and a 7 mm span length. Mechanical properties of the entire femur, such as ultimate force, maximum displacement, stiffness, and whole bone toughness, were assessed by analyzing load-displacement diagrams.

### BMSC culture

Primary bone marrow stromal cells (BMSC) were isolated from femurs of 3-month-old male mice as previously described.^75^ Briefly, bone marrow cells were cultured on 60-mm cell culture dishes in α-MEM (Hyclone, USA) containing 15% FBS. After 48 h, the non-adherent cells were removed. On the seventh day, the cells trypsinized for subsequent experiments. Colony forming unit-fibroblast (CFU-F) assay and colony forming unit-osteoblast (CFU-OB) assay were performed as we previously described.^28^

### ELISA assay

Serum levels of P1NP were measured using the RatLaps EIA Kit (cat# AC-33F1) according to the manufacturer’s instruction (Immunodiagnostic Systems Limited). Serum levels of CTX, degradation products from type I collagen during osteoclastic bone resorption, were measured using the RatLaps EIA Kit (cat# AC-06F1) according to the manufacturer’s instruction (Immunodiagnostic Systems Limited).

### Cell adhesion assay

MLO-Y4 cells were seeded in 96-well plates at a concentration of 1 × 10^5^ cell/mL, followed by a range of 5 dilutions, the cell number is 1 × 10^5^, 0.5 × 10^5^, 0.25 × 10^5^, 0.125 × 10^5^. CCK8 solution was added to 96-well plastic plates incubate for 1 h, and the optical density (OD) was measured to establish a standard curve for cell counts (cell number to OD). Then, 1 × 10^5^ control and knockdown cells were seeded in 96-well plates and allowed to attach to the substrate for different time points (0.5, 1, 3, 6, and 12 h), followed by three times of 1×PBS extensive wash to remove non-attached cells. Subsequently, CCK8 solution was added to each well and incubate for 1 h. Then the OD values of each well were assessed to calculate the cell counts in each well. The adhesive cell proportion was determined based on the calculated percentage of adhesive cells regarding to the seeding cell amount.

### RNA extraction and qRT-PCR analysis

RNA isolation and quantitative real-time RT-PCR analysis were performed as we previously described.^37^ The specific primers for gene expression analysis were listed in Table S1.

### Western blot analysis

Western blot analysis was performed as previously described.^76^ Briefly, whole-cell lysates were prepared in RIPA lysis buffer (Sigma, USA) and aliquots protein were separated by SDS-PAGE and blotted onto a polyvinylidene fluoride (PVDF) membrane (Millipore, MA, USA). Membranes were blocked at room temperature for 15 min in QuickBlock™ Western (Beyotime), followed by an overnight incubation at 4 °C with specific antibodies. After incubation with appropriate HRP-conjugated secondary antibodies (ZSGB-bio), blots were developed using an enhanced chemiluminescence (ECL Kit, BIORAD) and exposed in ChemiDoc XRS chemiluminescence imaging system. Antibodies used in this study are listed in Table S2.

### CRISPR/Cas-9 technology

The methodology described by Ran et al was employed to perform CRISPR/Cas-9 deletion of the vinculin expression in MLO-Y4 cells.^77^ The Vcl-sgRNA-GFP plasmid was transfected into MLO-Y4 cells. GFP-positive cells were sorted via flow cytometry and re-distributed as single cell clone across five 96-well plates. KD-1 and KD-2 cells were selected as representative clones for Vcl knock-down cell lines. The sgRNAs used in CRISPR/Cas9 deletion of mouse vinculin in this research are provided in the Table S3.

### Co-immunoprecipitation (Co-IP) assay

Co-IP assay was performed as previously described.^23^ Briefly, cells were incubated for 10 min at 4 °C in RIPA buffer (Sigma, USA). After a centrifugation at 12 000 × *g* for 10 min at 4 °C, the supernatant was first incubated with corresponding primary antibody overnight and then with Protein A/G Magnetic Beads at room temperature for 1 h. DynaMag™-2 Magnet (Thermo Fisher) was used to collect dynabeads-antigen-antibody complex. The complex was washed with IP buffer three times and resuspended with 60 μL 1× loading buffer and cooked at 95 °C for 5 min, followed by SDS-PAGE and western blotting.

### Chromatin immunoprecipitation (ChIP) assay

ChIP assay was performed using the ChIP Kit (Abcam, ab500) as previously described.^78^ Briefly, 1 × 10^7^ cells were collected in lysis buffer and sonicated to generate chromatin samples with average fragment sizes of 100–500 bp. Cell lysates were incubated with the indicated antibodies or IgG overnight at 4 °C. Then, the supernatants were mixed with the blocked Protein A/D Sepharose beads to collect the antibody–chromatin complexes. After washing 4 times, the immunoprecipitated DNA was eluted and purified for the subsequent qPCR analysis.

### Three point bending (3PB) test

Fresh femurs were dissected free of soft tissue and immediately kept in ice-cold 1x PBS for 3PB test. The strength test was performed at the midshaft of femurs by ElectroForce (Bose ElectroFore 3200; EndureTEC Minnetonka, MN, USA) with continuous displacement of 0.05 m/s in a single stop setting (Ramp waveform, span length, 7 mm). Whole femur mechanical properties, including ultimate force and whole bone toughness, were determined using load-displacement diagrams.

### Statistical analyses

The sample sizes for all experiments conducted in this study were determined based on our previous experience on similar studies. Mice used in this study were randomly grouped. The IF, IHC and histology were conducted and analyzed under double-blind conditions. Statistical analyses were completed using the Prism GraphPad. Two-way ANOVA and unpaired two-tailed Student’s *t* test was used as indicated. *P* < 0.05 was considered statistically significant.

## Supplementary information

Supplementary Figure

Supplementary Figure legend

Supplemental Information

## Data Availability

All data generated or analyzed during this study are included in the manuscript and supporting file.
