# Supplementary material for: Osteocytic vinculin controls bone mass by modulating Mef2c-driven sclerostin expression in mice
Source: Bone Res. 2025 Aug 13;13:73. doi: 10.1038/s41413-025-00452-x (PMC12343990; doi:10.1038/s41413-025-00452-x)

Supplementary Figure 1

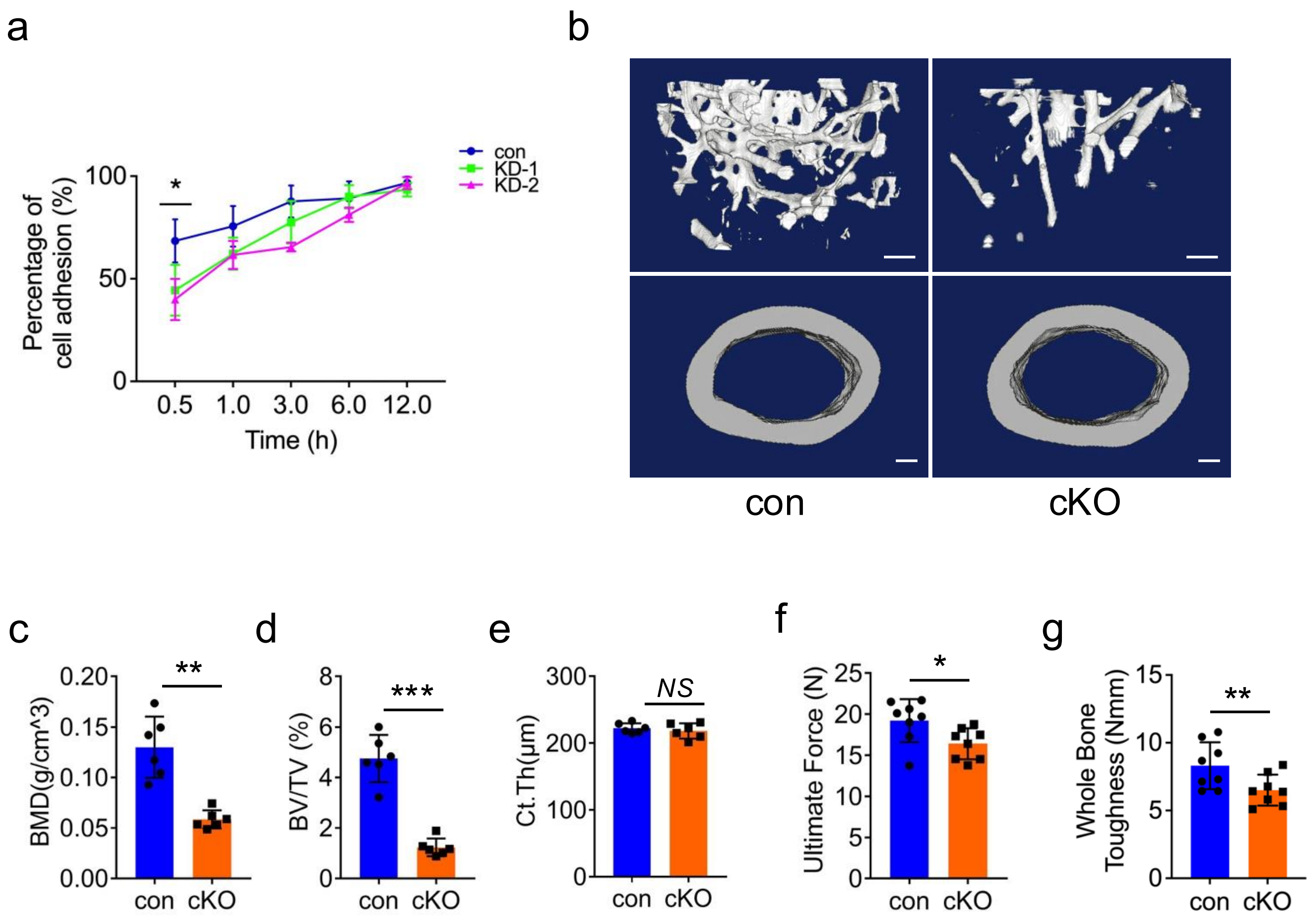

Supplementary Figure 2

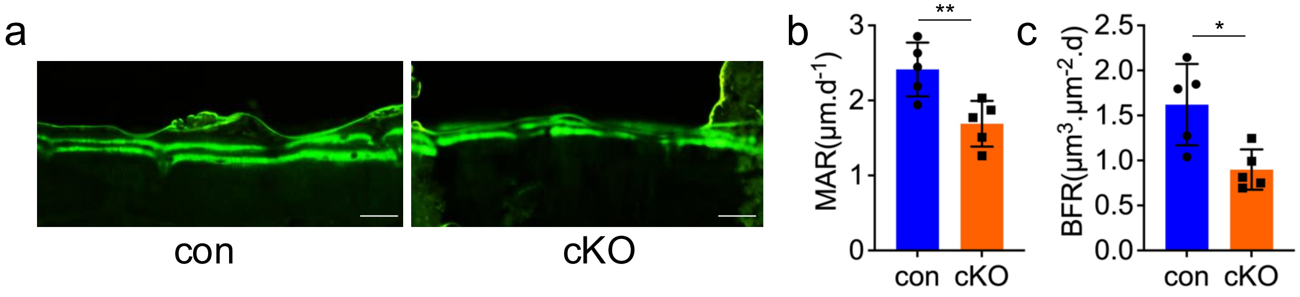

Supplementary Figure 3

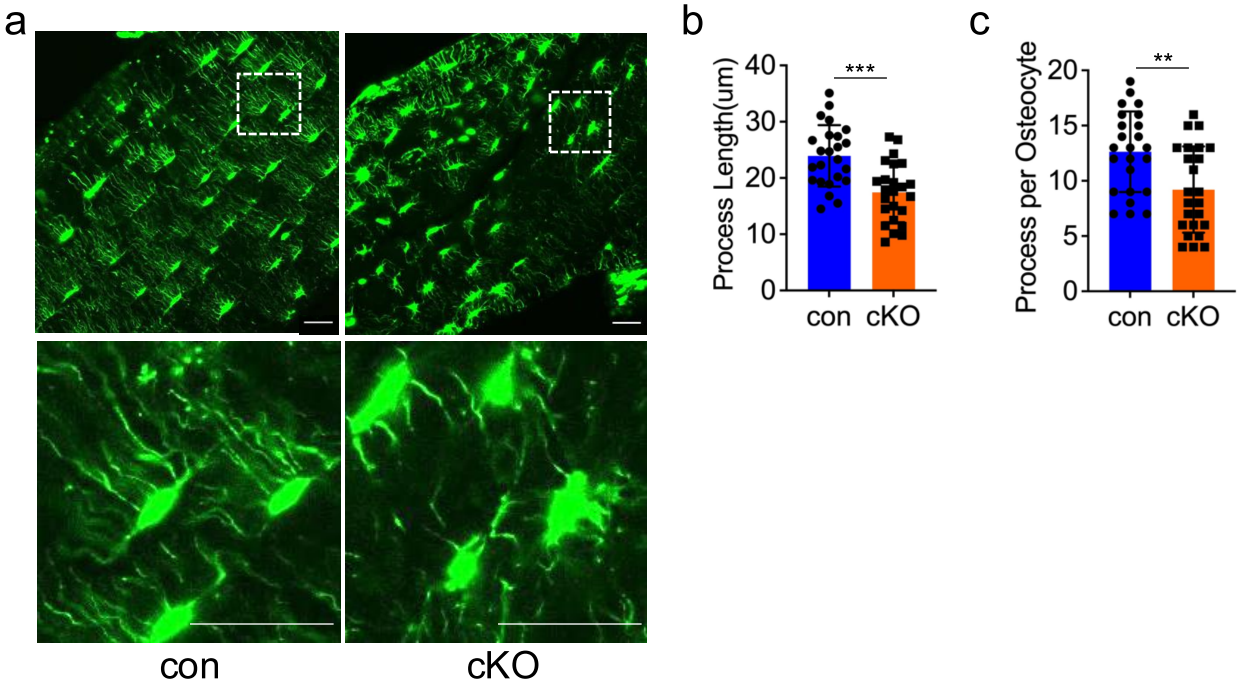

Supplementary Figure 4

a

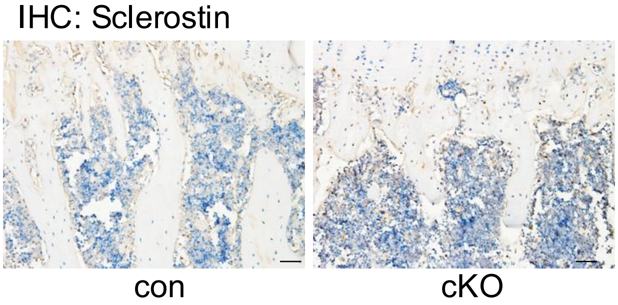

b

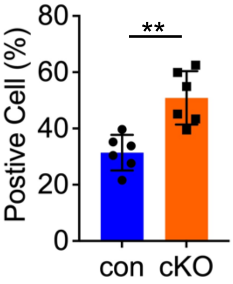

Supplementary Figure 5

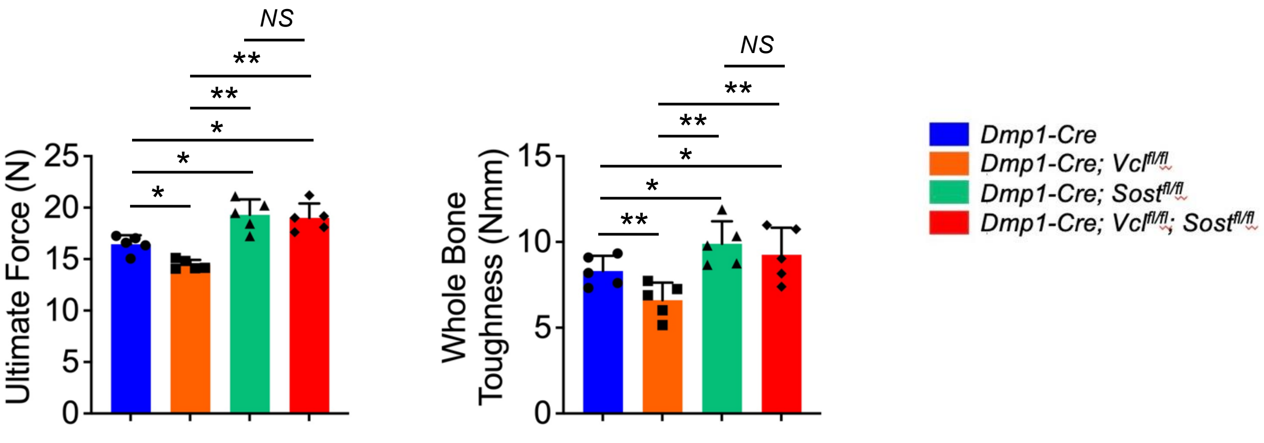

Supplementary Figure 6

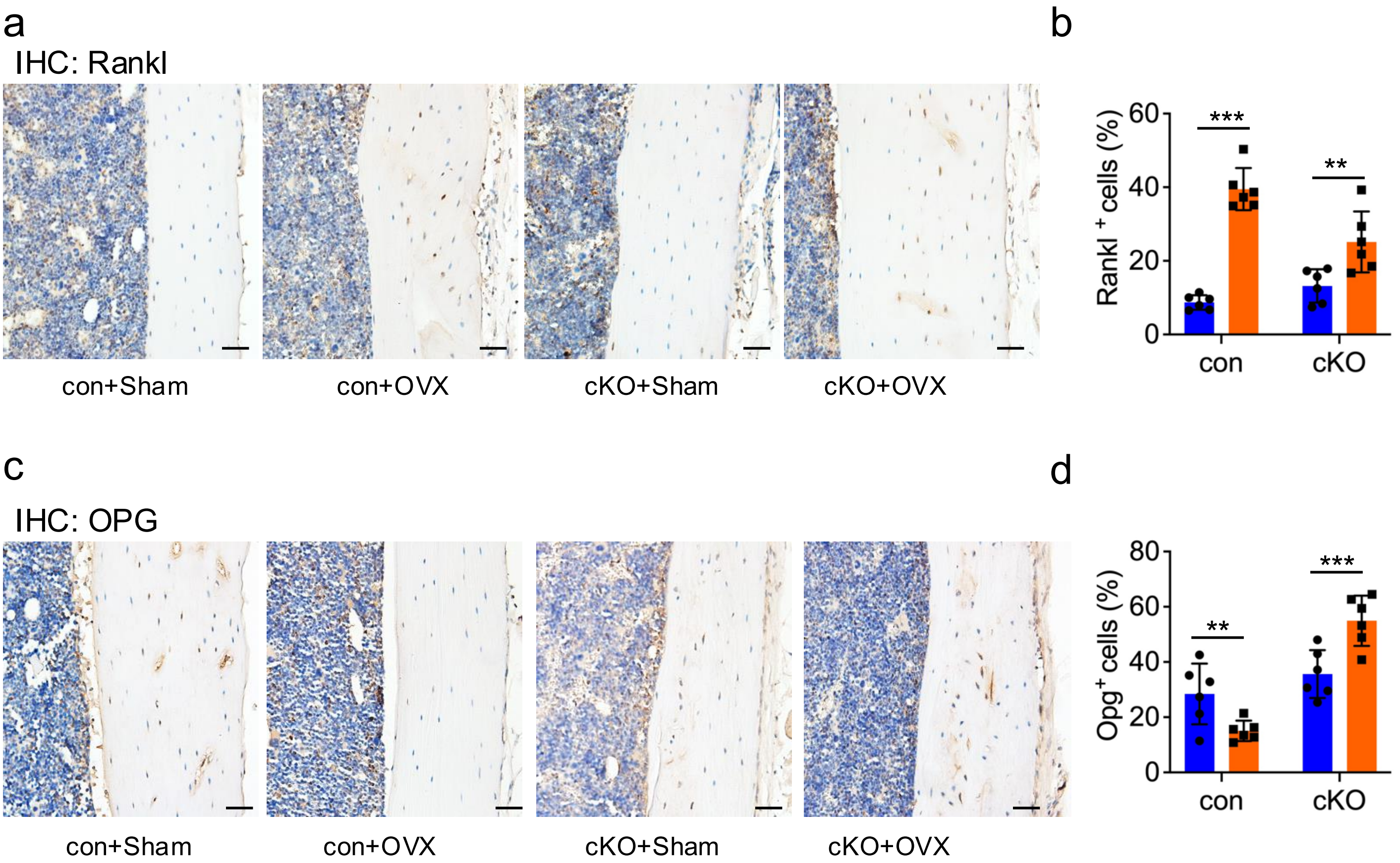

Supplement: Supplementary file 1 — Supplementary Figure [file 41413_2025_452_MOESM1_ESM.pdf]
