# Supplementary material for: Osteocytic vinculin controls bone mass by modulating Mef2c-driven sclerostin expression in mice
Source: Bone Res. 2025 Aug 13;13:73. doi: 10.1038/s41413-025-00452-x (PMC12343990; doi:10.1038/s41413-025-00452-x)
Supplement: Supplementary file 2 — Supplementary Figure legend [file 41413_2025_452_MOESM2_ESM.pdf]

**Supplementary Figure 1. Vinculin loss in Dmp1-expressing cells causes severe osteopenia in female mice**. **a** The percentages of cell adhesion on Col-I coated surface. MLO-Y4 cells with and without vinculin KD were seeded in a collagen coating 96-well plate at a density of $1\times10^5$ cells/well. The absorbance was measured at the time points of 0.5h, 1h, 3h, 6h and 12h, respectively. Adhesion assays for each time points were repeated for three times. **b** 3D reconstruction from micro-computerized tomography (μCT) scans of distal femurs from 3-mo- female control and cKO mice. Scale bar, 250 mm. **c-e** Quantitative analyses of the bone volume/tissue volume (BV/TV), cortical thickness (Ct.Th) and bone mineral density (BMD) of distal femurs from 3-mo-old female control and cKO mice. Results were expressed as mean ± s.d., $N = 6$ biologically independent replicates per group. **f, g** Quantitative analyses of ultimate force and whole bone toughness in three-point-bending test with from 3-mo-old male mice. $N = 8$ for control and cKO group, $^*P < 0.05$, $^{**}P < 0.01$, $^{***}P < 0.001$ versus controls, unpaired two-tailed Student's *t* test.

**Supplementary Figure 2. Vinculin loss in mature osteoblasts and osteocytes induces impaired bone formation in cortical bone. a-c** Calcein double labeling. Representative images of femur sections (a). Sections were used for measurements of MAR and BFR for metaphyseal femur bones. Scale bar, 50 mm. Results were expressed as mean ± s.d., $N = 5$ biologically independent replicates per group, $^*P < 0.05$, $^{**}P < 0.01$ versus controls, unpaired two-tailed Student's *t* test.

**Supplementary Figure 3. The formation of osteocyte dendrites is dependent on vinculin. a** Representative actin cytoskeleton images from Rhodamine-Phalloidin staining of the cortical femur sections from 6-month-old male control and cKO mice. Scale bar, 20 mm. **b, c** Quantitative analyses of cortical osteocyte process length and number for control and cKO mice. Results were expressed as mean ± s.d., $N = 5$, $*P < 0.05$, $**P < 0.01$ versus controls, unpaired two-tailed Student's *t* test.

**Supplementary Figure 4. Sclerostin-positive cells is increased in cortical bones of cKO mice as compared to the control group.**

**a-b** Immunohistochemical (IHC) staining of tibial sections from 3-mo-old control and cKO male mice treated with sclerostin antibody(a). Quantitative data (b) Scale bar, 50 mm. $**P < 0.01$ versus controls, unpaired two-tailed Student's *t* test.

**Supplementary Figure 5. Sclerostin deficiency in mature osteoblasts and osteocytes reverses the mechanical properties induced by vinculin loss.** a, b Quantitative analyses of ultimate force and whole bone toughness in three-point-bending test with from 2.5-mo-old male mice. $N = 5$ for each group, $*P < 0.05$, $**P < 0.01$ versus controls, two-way ANOVA.

**Supplementary Figure 6. Vinculin loss in Dmp1-expressing cells changes the ratio of Rankl/Opg in osteocytes. a-d** Immunohistochemical (IHC) staining of tibial sections from 5-mo-old control and cKO female mice performed with sham or OVX

surgeries. Samples treated with an antibody against Rankl (a) or OPG (c). Quantitative data (b, d) Scale bar, 50 mm. Results were expressed as mean ± s.d., $N = 6$ biologically independent replicates per group, $*P < 0.05$, $**P < 0.01$ versus controls, unpaired two-tailed Student's $t$ test.
