# Supplementary material for: Osteocytic vinculin controls bone mass by modulating Mef2c-driven sclerostin expression in mice
Source: Bone Res. 2025 Aug 13;13:73. doi: 10.1038/s41413-025-00452-x (PMC12343990; doi:10.1038/s41413-025-00452-x)
Supplement: Supplementary file 3 — Supplemental Information [file 41413_2025_452_MOESM3_ESM.pdf]

Supplementary information

Focal adhesion (FA)

Extracellular matrix (ECM)

Dentin matrix protein 1 (Dmp1)

Institutional Animal Care and Use Committees (IACUC)

Micro-computerized tomography (μCT)

American Society for Bone and Mineral Research (ASBMR)

Faraformaldehyde (PFA)

Hematoxylin and eosin (H/E)

Tartrate-resistant acid phosphatase (TRAP)

Immunohistochemistry (IHC)

Immunofluorescence (IF)

Bone marrow stromal cells (BMSC)

Colony forming unit-fibroblast (CFU-F)

Colony forming unit-osteoblast (CFU-OB)

Polyvinylidene fluoride (PVDF)

Co-immunoprecipitation (Co-IP)

Chromatin immunoprecipitation (ChIP)

Knocked down (KD)

Bone mineral density (BMD)

Bone volume fraction (BV/TV)

Cortical thickness (Ct.Th)

Mineralization apposition rate (MAR)

Bone formation rate (BFR)

Procollagen type 1 amino-terminal propeptide (P1NP)

Osteoid volume/tissue volume (OV/TV)

Bone volume/tissue volume (BV/TV)

Collagen type I cross-linked C-telopeptide (CTX)

Osteoclast surface/bone surface (Oc.S/BS)

Osteoclast number/bone perimeter (Oc.Nb/BPm)

Ovariectomy (OVX)

Supplementary Table 1: Mouse real-time RT-PCR (qPCR) primers

Name	5’ primer	3’ primer
Vcl	GCTTCAGTCAGACCCATACTCG	AGGTAAGCAGTAGGTCAGATGT
Gapdh	CAGTGCCAGCCTCGTCCCGTAG	CTGCAAATGGCAGCCCTGGTGAC
Sost	AGCCTTCAGGAATGATGCCAC	CTTTGGCGTCATAGGGATGGT

Supplementary Table 2: Antibody information

Antibody	Company	Catalog #	Application/Dilution
Mef2c	Proteintech	10056-1-AP	WB (1:2000) IF (1:200)
talin-1	Proteintech	14168-1-AP	WB (1:1000) IF (1:200)
vinculin	Proteintech	66305-1-Ig	WB (1:1000) IF (1:200)
active β-catenin	CST	8814S	WB (1:2000)
β-catenin	CST	9562S	WB (1:1000)
lamin b1	CST	13435s	WB (1:1000)
Flag-Tag	CST	14793S	WB (1:1000)
HA-Tag	CST	3724S	WB (1:1000)
pinch1	Abcam	ab108609	WB (1:1000), IF (1:200)
β1 integrin	Abcam	ab95623	WB (1:1000), IF (1:200)
sclerostin	Abcam	Ab63097	WB (1:1000), IHC (1:200)
phalloidin-488	Invitrogen™	A12379	IF (1:500)
tubulin	ZSGB-BIO	TA-10	WB (1:1000)
Gapdh	ZSGB-BIO	TA-08	WB (1:1000)

Supplementary Table 3: vinculin sgRNA information

vcl-sgRNA1	5’- CGGCCGTCAGCAACCTCGTCCGG-3’
vcl-sgRNA2	5’-GCTACGGGCGCGGTGAGGTCAGG-3’
